# Endoplasmic reticulum stress aggravates ferroptosis via PERK/ATF4/HSPA5 pathway in UUO-induced renal fibrosis

**DOI:** 10.3389/fphar.2025.1545972

**Published:** 2025-04-04

**Authors:** Zhigang Huang, Lihua Zhou, Bin Liu, Xiaoju Li, Yu Sang

**Affiliations:** ^1^ Department of Urology, The First Affiliated Hospital of Yangtze University, Jingzhou, China; ^2^ Hubei Key Laboratory of Medical Technology on Transplantation, Zhongnan Hospital of Wuhan University, Institute of Hepatobiliary Diseases of Wuhan University, Transplant Center of Wuhan University, National Quality Control Center for Donated Organ Procurement, Hubei Provincial Clinical Research Center for Natural Polymer Biological Liver, Wuhan Hubei, China; ^3^ Department of Traditional Chinese Medicine, The First Affiliated Hospital of Yangtze University, Jingzhou, China

**Keywords:** HSPA5, ferroptosis, endoplasmic reticulum stress, epithelial-tomesenchymal transition, renal fibrosis

## Abstract

Renal fibrosis, resulting from the transformation of damaged tubular epithelial cells (TECs), serves as a prevalent pathological condition observed in nearly all forms of advancing chronic kidney disease (CKD). Although crucial in fibrotic diseases, the association between endoplasmic reticulum stress (ERS) and ferroptosis remains incompletely elucidated. Herein, increased levels of heat shock protein family A member 5 (HSPA5), acting as a co-molecular in ERS and ferroptosis, along with EMT-associated alterations, including increased α-smooth muscle actin (α-SMA) and Col1a1 levels and decreased E-cad expression, were observed in fibrotic kidneys of Unilateral Ureteral Obstruction (UUO)-induced mouse models and TGF-β-induced EMT in HK-2 cells. The employment of ferrostatin-1 (Fer-1) improved these alterations and reversed TGF-β-induced EMT *in vitro*. More importantly, Inhibiting ERS by Tauroursodeoxycholate (TUDCA) reversed the alterations of ferroptosis, including GPX4 expression, reactive oxygen species (ROS) accumulation, iron overload, increased lipid peroxidation production, as well as EMT progression *in vivo* and *in vitro*. Whereas the overexpression of HSPA5 strikingly attenuated the inhibitory effects of TUDCA on ferroptosis and TGF-β-induced EMT *in vitro*. Mechanistically, Co-immunoprecipitation (Co-IP) tests showed that ATF4 engaged with and SUMOylated HSPA5 to trigger the HSPA5 signaling pathway in response to TGF-β. These findings illuminate that focusing on HSPA5 may present a promising therapeutic approach to enhance tubular epithelial cells’ survival and alleviate the progression of CKD.

## 1 Introduction

Renal fibrosis is a prevalent pathological condition observed in almost all types of progressive chronic kidney disease (CKD) (Duffield, 2014; Humphreys, 2018). It is manifested by interstitial fibrosis, tubular atrophy (IF/TA), inflammatory infiltration within fibroblast proliferation, and excessive deposition of extracellular matrix (ECM) (Humphreys, 2018). Tubular epithelial cells (TECs), considered among the primary cell types in kidney responding to injuries, have been identified as initiators of human kidney fibrosis rather than merely victims of the process (Kriz et al., 2011). There is growing evidence indicating that injured TECs undergo a partial epithelial-to-mesenchymal transition (EMT) while retaining their location within the tubular basement membrane to acquire mesenchymal characteristics, subsequently leading to an increase in the inflammatory response and fibrogenesis (Nastase et al., 2018; Panizo et al., 2021). Hence, it is crucial to investigate the mechanisms driving EMT to uncover novel strategies for CKD.

Ferroptosis is a newly form of cell death controlled by the lipid-repair enzyme glutathione peroxidase 4 (GPX4) and induced by pronounced lipid peroxidation, dependent on reactive oxygen species (ROS) generation and excess of iron (Jiang et al., 2021). Despite recent research indicating the importance of ferroptosis in different kidney conditions such as renal cell carcinoma, AKI, and DN, the understanding of ferroptosis in CKD is still limited (Belavgeni et al., 2020; Chen et al., 2021; Xie et al., 2022; Wang et al., 2020), exploration of ferroptosis in CKD remains incompletely defined. Of note, excessive accumulation of iron in the tubules was a significant factor in the deterioration of kidney damage, leading to higher levels of ROS and inflammation in various forms of CKD (Li et al., 2022). Interestingly, iron metabolism significantly influences the EMT process, with highly mesenchymal cells demonstrating increased susceptibility to ferroptosis (Oliveira et al., 2021). However, the connection between ferroptosis and renal fibrosis, along with the controlling function of ferroptosis in renal partial EMT, is mostly unexplored.

The heat shock protein family A member 5 (HSPA5), commonly known as 78 kDa glucose-regulated protein (GRP78), played a pivotal role in various human ailments, including infections and tumorigenesis (Wang Q. et al., 2023; Ibrahim et al., 2019). The abnormal overexpression of HSPA5 drived the advancement of breast cancer, liver cancer and various other cancer types (Yao et al., 2020; Zhu et al., 2017). Chen et al. showed that DHA elevated GPX4 expression through HSPA5 upregulation, providing cellular protection against ferroptosis in pancreatic cancer cells (Zhu et al., 2017), mirroring observations in glioma cells (Chen et al., 2019). In colorectal cancer, HSPA5 suppresses ferroptosis in tumor cells to enhance cancer progression by preserving GPX4 stability (Wang et al., 2022). Nevertheless, the involvement of HSPA5-mediated ferroptosis in kidney fibrosis has not been documented, and the mechanism behind it is still unknown.

Endoplasmic reticulum stress (ERS) was essential in promoting EMT advancement in fibrotic diseases and cancers. Upon ERS initiation, protein kinase R (PKR)-like ER kinase (PERK) was activated, causing eukaryotic initiation factor 2α (eIF2α) to be phosphorylated, which resulted in an increase in activating transcription factor 4 (ATF4) (Chen and Cubillos-Ruiz, 2021; Sun et al., 2023). Several studies shown that HSPA5 enhanced tumor cell survival and conferred drug resistance during ERS (Wang Y. et al., 2023). Erastin-induced ferroptosis could raise ERS-associated ATF4 expression, resulting in an increase in SLC7A11 levels via a loop that counteracts ferroptosis in bladder cancer cells (Ye et al., 2014). Furthermore, inhibition of ERS markedly reduced the levels of iron and lipid peroxidation in colonic epithelial cells (Chang et al., 2018). Given the unique processes of ERS and ferroptosis in cancerous cells, we postulate a novel theory whereby ERS impacts ferroptosis in TECs during EMT in renal fibrosis.

This study examined the mechanism by which ERS controls ferroptosis and explores the relationship between ERS and HSPA5, providing a theoretical foundation for the treatment of CKD patients.

## 2 Materials and methods

### 2.1 Data retrieval and organization

RNA sequences from CKD samples were retrieved from the GEO database (GSE66494, Control group: 8, CKD group: 53). Differential genes were extracted from both CKD and control group samples in the GEO database. By utilizing the ferroptosis-associated genes listed on the FerrDb website (http://www.zhounan.org/ferrdb/current/), we identified ferroptosis-related differential genes. Subsequently, the overlapping differential genes from both datasets were identified for further analysis. This process was conducted by two expert bioinformatics analysts. Data analysis was carried out using the R4.2.2 software.

### 2.2 Construction of prediction model

Lasso regression analysis was conducted to identify more representative genes from the differentiated iron death-related genes, followed by Logistic regression analysis to identify potential predictive genes. Potential prognostic genes were identified based on their statistical significance (p-value <0.05) in the logistic regression analysis. Patients were categorized into low-risk and high-risk categories based on their risk score, with the median score used as the cutoff point. Subsequently, we validated the accuracy of the prediction model using ROC curve analysis. Furthermore, the disparities in modeled gene expression were visualized through scatter plots.

### 2.3 Functional enrichment analysis

We used the “clusterProfiler” and “org.Hs.e.g.,.db” packages in R to examine the pathways of the genes with differential expression for Gene Ontology (GO) and Kyoto Encyclopedia of Genes and Genomes (KEGG), while also performing analysis on differential gene pathways and functional enrichment. Moreover, we investigated the Protein-Protein Interaction (PPI) network to analyze the connections between various differentially expressed genes using the website (https://cn.string-db.org/). After analyzing the pathways with varying expression levels in high and low-risk groups, we pinpointed the pathways that showed significant differences in expression levels between the high and low expression groups. These findings helped direct the next steps in the study after setting up filtering criteria.

### 2.4 Animal treatment

Adult male C57BL/6J mice weighing 22–30 g were housed at Yangtze University’s Laboratory Animal Center (Jingzhou, China). The experimental procedures were approved by the Ethics Committee of Yangtze University (animal ethical committee number KY202434) and followed the guidelines outlined in the Care and Use of Laboratory Animals. The mice were given unrestricted access to food and water in a controlled, pathogen-free setting with the right temperature and humidity levels. The method of unilateral ureteral obstruction (UUO)-induced fibrosis model was outlined in the prior experiments (Masaki et al., 2003). In summary, C57BL/6 mice were anesthetized via inhalation with isoflurane, and an incision in the mid-abdominal area was made to access the left kidney, along with the left renal artery, vein, and ureter. Following this, the top portion of the left ureter was tied twice with 4–0 silk sutures and then incised between the ties in the UUO group. The Tauroursodeoxycholate (TUDCA) group were administered intraperitoneal injections of 150 mg/kg/day TUDCA (HY-19696, MedChemExpress, United States) dissolved in PBS before UUO surgery. Six mice from each experimental group were euthanized on the 7th and 14th days post-surgery. Left kidney tissues were collected for standard measurements and subsequent experiments.

### 2.5 Cell treatment and reagents

The HK-2 cells were obtained from the American Type Culture Collection (ATCC, Manassas, Virginia). To summarize, cells were grown in DMEM/F12 medium (Invitrogen, United States) with 10% FBS and 1% penicillin-streptomycin (Gibco, United States) at 37°C in 5% CO2. HK-2 cells underwent stimulation with transforming growth factor-β (TGF-β, 10 ng/mL, PeproTech) for 24 h to induce Epithelial-Mesenchymal Transition (EMT). Moreover, prior to TGF-β stimulation, cells in certain experimental groups were pre-exposed to Ferrostatin-1 (Fer-1) (1–100 μM, HY-100579, MedChemExpress, United States) or TUDCA (200 μM, HY-19696, MedChemExpress, United States).

### 2.6 *In vitro* transfection

HSPA5 or ATF4 knockdown was achieved in HK-2 cell lines by transfecting HSPA5 siRNA (sense 5′-UUU​UCA​ACC​ACC​UUG​AAC​GGC-3′ and antisense 5′-CGU​UCA​AGG​UGG​UUG​AAA​AGA-3′) or ATF4 siRNA (5′-UGU​CUU​UGU​CGG​UUA​CAG​CAA-3′ and antisense 5′-GCU​GUA​ACC​GAC​AAA​GAC​ACC-3′) obtained from KeyGen BioTECH (Nanjing, Jiangsu, China) using Lipofectamine 3,000 (Thermo Fisher Scientific). Initially, HK-2 cells were plated in six-well plates. Upon reaching confluence, the cells were serum-deprived for 24 h, followed by preincubation with siRNA targeting HSPA5 or ATF4 for 8 h. Subsequently, the cells were exposed to TGF-β stimulation for 24 h. The efficacy of siRNA inhibition was confirmed through Western blot analysis.

Flag-ATF4-lentivirus and HA-HSPA5-lentivirus plasmids from GeneChem (Shanghai, China) were used to create HEK-293T cell lines with increased levels of HSPA5 and ATF4 through transfection. HEK-293T cells were transfected separately with the plasmids and incubated for 24 h before undergoing puromycin selection at a concentration of 6 μg/mL (Beyotime, Shanghai, China).

### 2.7 Cell viability assay

Cell viability was assessed utilizing an MTT assay kit (Thermo Fisher Scientific, United States). HK-2 cells were seeded into 96-well plates and incubated for 1 day. Subsequently, the cells were exposed to varying concentrations of Fer-1. Each well was supplemented with a new medium containing 0.5 mg/mL MTT solution and then incubated at 37°C for 4 h. The VersaMax ELISA Microplate Reader (Molecular Devices, Silicon Valley, CA, United States) was utilized to measure absorbance at 570 nm. Each reaction was repeated five times.

### 2.8 Western blot (WB) and immunoprecipitation (IP) analysis

WB and IP experiments were conducted as previously outlined (Kang et al., 2001). To summarize, the protein specimens were subjected to 10% SDS-PAGE gels, followed by transfer onto PVDF membranes, and subsequent blocking for 30 min with Protein-Free Rapid Blocking Buffer (Epizyme, Shanghai, PS108P). Following this, they were exposed to primary antibodies and left to incubate overnight at 4°C, including GPX4 (1:1,000, ab125066, Abcam), HSPA5 (1:1,000, ab21685, Abcam), Col1a1 (1:1,000, A1352, Abclonal), α-SMA (1:500, #19245, CST), E-cad (1:1,000, 40,860, SAB), ATF4 (1:500, 10835-1-AP, Proteintech), CHOP (1:1,000, 15204-1-AP, Proteintech), PERK (1:1,000, # 3,192, CST), p-PERK (1:1,000, 29546-1-AP, Proteintech), SUMO1(1:1,000, ab32058, Abcam), GAPDH (1:1,000, 60004-1-Ig, Proteintech). The next day, they were exposed to secondary antibodies against mouse or rabbit (1:5,000 dilution, SA00001-1, SA00001-2 Proteintech) at ambient temperature for 1 h. ImageJ software (NIH, Bethesda, MD, United States) was utilized to calculate band densities.

### 2.9 Measurement of malondialdehyde (MDA), glutathione (GSH), and iron level

An MDA detection kit (DOJINDO, M496) was employed for measuring MDA content, and a GSH/GSSG detection kit (Sigma-Aldrich) was utilized for assessing GSH/GSSG levels following the manufacturer’s protocols. Intracellular overall iron content was evaluated following the manufacturer’s guidelines using an iron assay kit (Sigma-Aldrich).

### 2.10 Lipid peroxidase assay

Lipid peroxidation was assessed via fluorescence microscopy with the C11-BODIPY 581/591 probe (Thermo Fisher, D3861). HK-2 cells were placed in 6-well plates, treated with TGF-β, Fer-1, or TUDCA for 24 h, and then labeled with 5 μM C11-BODIPY using a fluorescence microscope. Afterward, the cells were placed in darkness and kept at a temperature of 37°C for half an hour. The cells were then rinsed twice with PBS, fixed in 4% paraformaldehyde, and imaged using a fluorescence microscope (Leica, Thunder Imager).

### 2.11 Histology and histopathology

Kidney specimens were preserved in 4% paraformaldehyde, encased in paraffin, and subsequently cut into slices that were 4 μm thick. Renal pathology was evaluated using Hematoxylin and eosin (H&E) staining, with collagen deposition visualized through Masson’s trichrome and Sirius red staining. Tubule injury was evaluated using a five-point grading system: 0 (normal), 1 (<25%), 2 (25–50%), 3 (50–75%), and 4 (≥75%). The tubulointerstitial fibrosis index was assessed by blinded experts and quantified using ImageJ software.

### 2.12 Immunohistochemistry and immunofluorescence staining

Immunohistochemical staining involved incubating 4-μm-thick tissue sections at 65°C for 2 h, deparaffinizing in xylene, rehydrating with a series of ethanol, performing antigen retrieval by boiling, and blocking with block buffer (Beyotime, P0260) for 30 min at room temperature. Afterwards, the samples were incubated overnight with primary antibodies at a temperature of 4°C. Target proteins, such as GPX4 (1:1,000, ab125066, Abcam) and HSPA5 (1:100, #3177, CST), were detected and examined by using primary antibodies during incubation with kidney sections, followed by secondary antibody treatment. Digital slide scanner (Leica, Aperio CS2) was used to scan the slides.

For immunofluorescence staining, the primary antibodies, such as HSPA5 (1:100, #3177, CST) and α-SMA (1:300, A5228, MilliporeSigma), were applied to the renal tissue sections and left to incubate overnight at 4°C. Goat Anti-Rat IgG H&L (Alexa Fluor^®^ 647) and Goat Anti-Mouse IgG H&L (Alexa Fluor^®^ 488) secondary antibodies from Abcam were used on day two at a dilution of 1:400. The proportion of positive staining was analyzed and quantified using ImageJ software.

### 2.13 Transmission electron microscopy

The kidney sections, fresh at the time, were sliced to a 2 mm thickness and the specimens were then placed in an electron microscope fixative for 2 h at room temperature before being stored at 4°C. Electron microscopy embedding and imaging services were provided by Servicebio Inc (Wuhan, China).

### 2.14 Statistical analysis

All data were presented as the mean ± SD of a minimum of three independent experiments. Statistical analyses were performed with GraphPad Prism software, version 7.0. Group comparisons for animals were evaluated using one-way analysis of variance (ANOVA) followed by either Dunnett’s test or Bonferroni correction as *post hoc* tests. P-value <0.05 was considered statistically significant.

## 3 Results

### 3.1 Acquisition of public differential genes and construction of the prediction model

**FIGURE 1:**
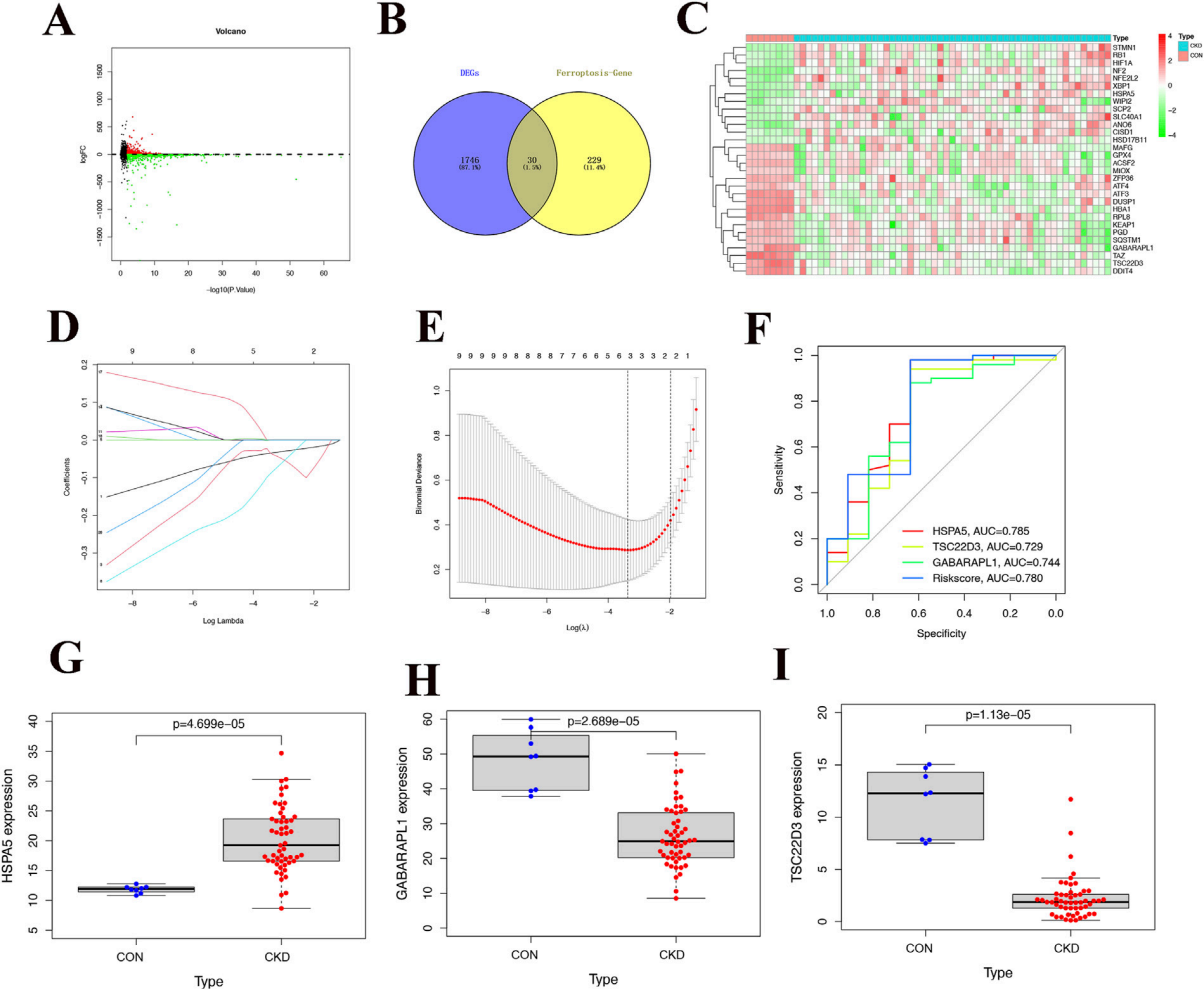
Acquisition of public differential genes and construction of the prediction model. **(A)** Volcano plot of differential genes in CKD samples, with red dots representing up-regulated differential genes and green dots representing downregulated differential genes. **(B)** Intersection of differentially expressed genes and ferroptosis-related genes, 30 ferroptosis-related differential genes. **(C)** Heat map of 30 common differentially expressed genes in CKD and normal tissues. **(D, E)** The Lasso regression analysis, it is evident that predicting with 3 genes yields higher accuracy and reliability. **(F)** ROC curve of different genes and Riskscore, the color red is associated with HSPA5, p < 0.05. **(G-I)** Differences in expression of 4 modeling genes between CKD and normal tissues.

Upon examination of CKD samples from the Gene Expression Omnibus (GEO), we discovered genes that were expressed differently between the control group samples and CKD (1776), as shown in Figure 1A. Subsequently, upon integrating the ferroptosis-related genes, a total of 30 common differential genes were obtained, with the findings presented in Figures 1B, C. LASSO regression analysis was conducted to identify the most representative genes, and the outcomes can be observed in Figures 1D, E. A one-way logistic regression analysis was used to discover possible prognostic genes, resulting in the creation of a prognostic model containing three CKD-related differential genes (HSPA5, TSC22D3, and GABARAPL1). Each sample underwent a risk assessment using a predictive model, resulting in the classification of patients as either low-risk or high-risk based on the median risk score cutoff. ROC curves illustrated the AUC values for the predicted genes: HSPA5 (AUC = 0.785), TSC22D3 (AUC = 0.729), and GABARAPL1 (AUC = 0.744) (Figure 1F). Notably, HSPA5 has notably emerged as the most accurate predictor of CKD and will be the primary focus of further investigation. The scatter plot analysis revealed elevated expression levels of HSPA5 in CKD tissues (Figure 1G). In contrast, the CKD tissues exhibited decreased expression levels of TSC22D3 and GABARAPL1 when compared to the control group (Figures 1H, I).

### 3.2 Functional enrichment analysis

**FIGURE 2:**
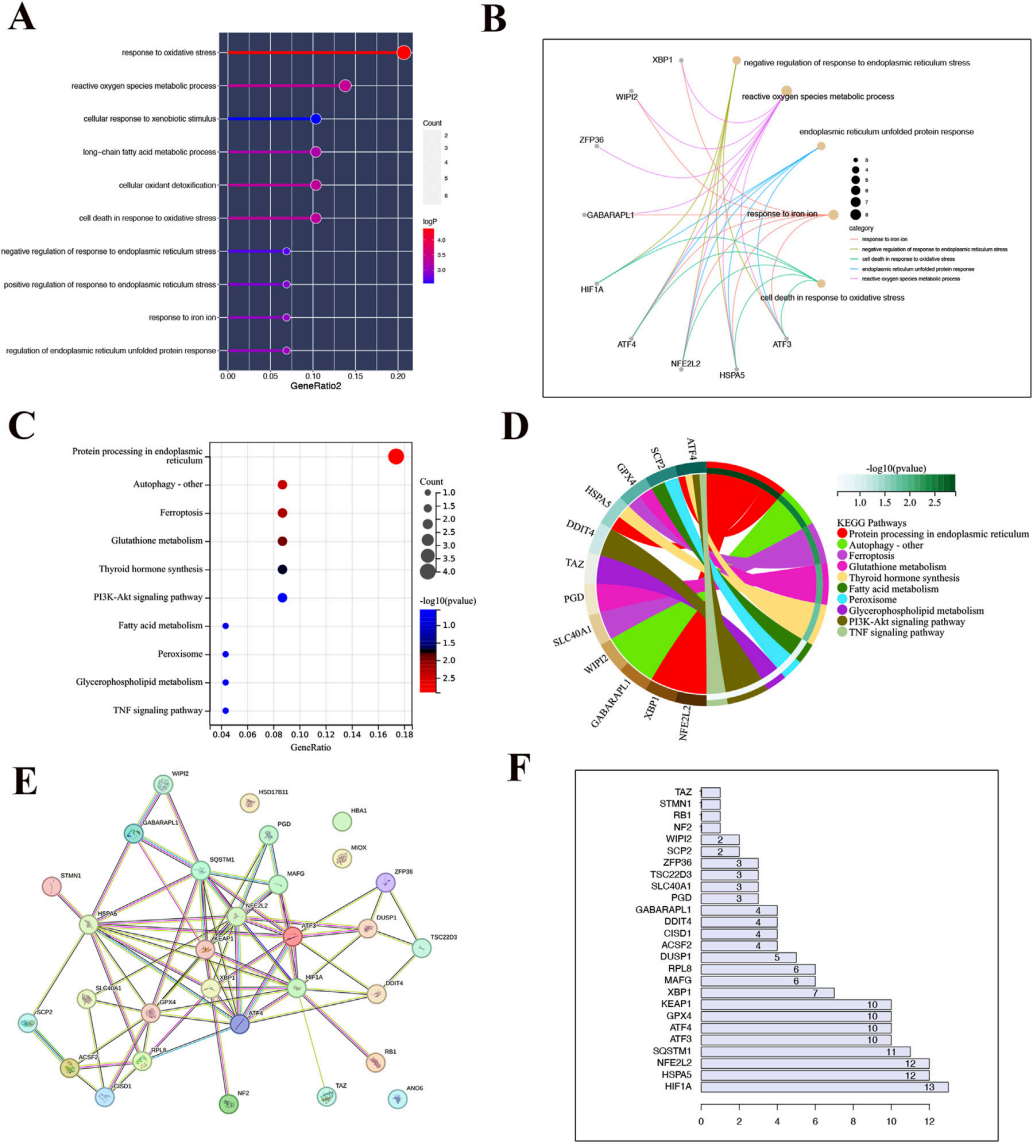
Functional enrichment analysis. **(A)** Bar chart of the result of GO enrichment analysis. **(B)** The point on the left half of the circle represents different genes, while the right half represents different pathways. The line connecting the two indicates that the gene is present in the pathway. **(C)** The outcome of KEGG enrichment analysis is displayed in a bar graph where the color indicates the P-value. A transition from light to dark colors signifies an increase in the P-value. The size of the endpoints on the graph corresponds to the number of genes enriched in the pathway, with larger endpoints indicating a higher number of enriched genes. **(D)** The point on the left half of the circle represents different genes, while the right half represents different pathways. The line connecting the two indicates that the gene is present in the pathway. **(E)** Results from the protein interaction network **(F)** Protein interaction statistics.

By conducting functional enrichment analysis, the GO enrichment analysis results (Figures 2A, B) revealed that the differential genes in CKD samples were predominantly concentrated in pathways related to oxidative stress, reactive oxygen species metabolism, cell death caused by oxidative stress, detoxification of cellular oxidants, metabolism of long-chain fatty acids, response to foreign substances, promotion and inhibition of endoplasmic reticulum stress response, iron ion response, and regulation of endoplasmic reticulum unfolded protein response. Additionally, to further explore the impact of these distinct genes on the progression of CKD, an analysis of KEGG enrichment was performed on the common differential genes. The outcomes revealed enrichment in pathways such as Protein processing in the endoplasmic reticulum, Autophagy, Ferroptosis, Glutathione metabolism, Thyroid hormone synthesis, Fatty acid metabolism, Peroxisome function, Glycerophospholipid metabolism, the PI3K-Akt signaling pathway. These results are depicted in Figures 2C, D. Additionally, analyses of the protein-protein interactions (PPI) highlighted HSPA5 as central to the interactions among these differential genes, as shown in Figures 2E, F.

### 3.3 Upregulated HSPA5, accompanied by ferroptosis was involved in the fibrotic kidneys of mice

**FIGURE 3:**
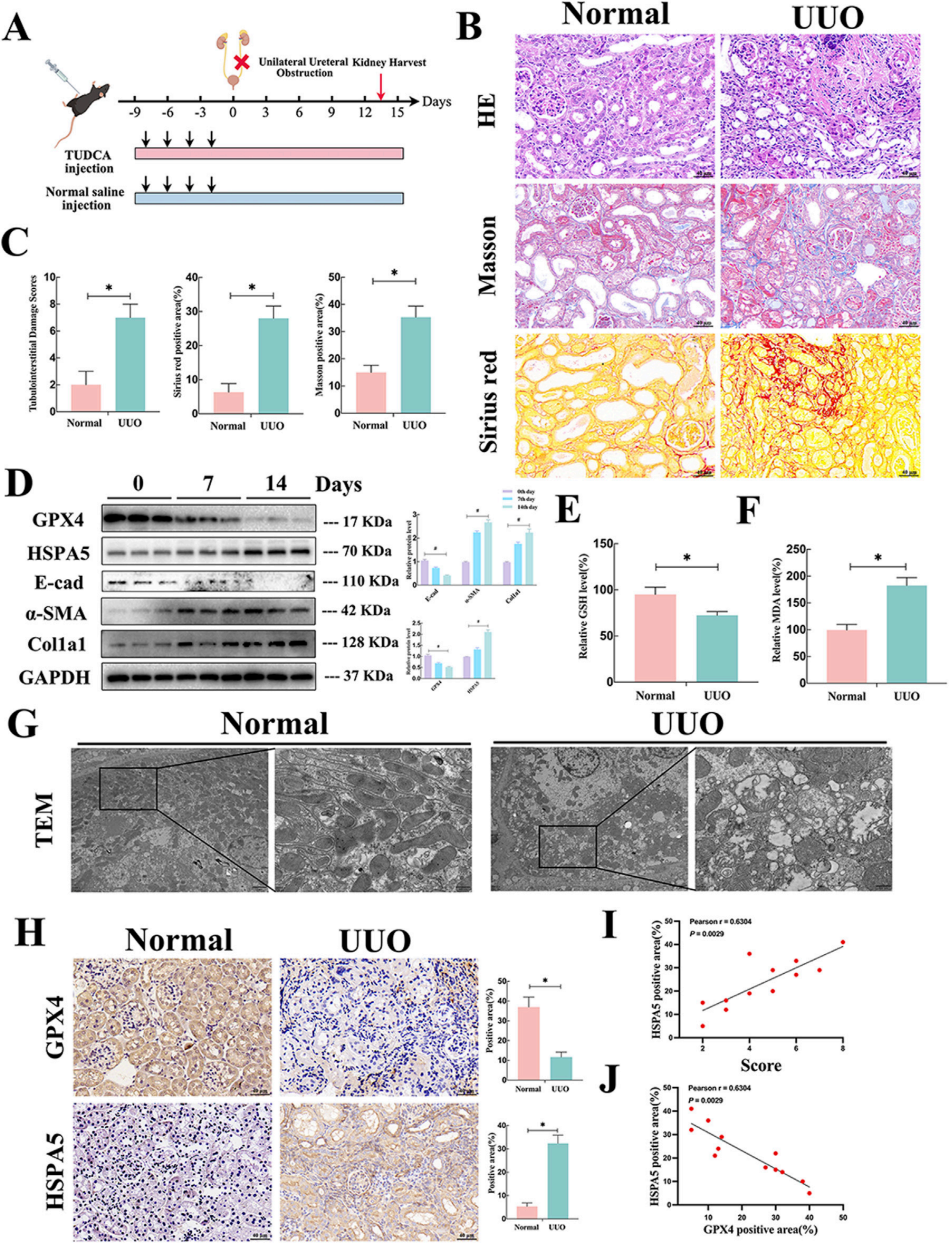
Upregulated HSPA5, accompanied by ferroptosis was involved in the fibrotic kidneys of mice. **(A)** Mouse experimental process depicted in a schematic diagram. **(B)** HE, Masson trichrome and Sirius red staining of kidney interstitium tissue of the 2 groups (Scale bar, 100μm, 20 μm). **(C)** Tubulointerstitial injury ratings and percentage of positive area were evaluated in various groups (n = 6, *P < 0.05) (n = 6, *P < 0.05). **(D)** Kidney lysates were collected and Western blot analysis was conducted. Representative blots of Col1a1, E-cad, α-SMA, GPX4 and HSPA5 (n = 6). **(E, F)** The GSH and MDA levels were detected in renal tissues from the normal and UUO groups (n = 6, *P < 0.05). **(G)** TEM analysis of RTECs in renal tissues from the normal and UUO groups. **(H)** Immunohistochemistry signals of GPX4 and HSPA5. Statistical analysis was conducted on the positive area of proteins in various groups (n = 6, *P < 0.05). **(I)** The relationship between HSPA5 levels and tubulointerstitial damage scores was analyzed (n = 12, Pearson χ2 test). **(J)** The correlation of HSPA5 expression and GPX4 expression (n = 12, Pearson χ2 test). The results are expressed as the mean ± standard error of the mean (S.E.M.).

To delve deeper into the role of HSPA5 and ferroptosis in mice, a renal fibrosis model was established through unilateral ureteral obstruction (UUO) (Figure 3A). In the UUO group, the H&E staining showed tubular atrophy with flattened epithelial cells, absence of brush border, and dilation of the lumen (Figures 3B, C). Additionally, interstitial extracellular matrix (ECM) deposition was observed through Sirius red and Masson staining (Figures 3B, C). Concurrently, the expression of profibrotic factors, including col1a1 and α-SMA, was increased in renal tissues at 7 and 14 days in the UUO group, concomitant with a reduction in E-cad expression as determined by Western blotting (Figure 3D). We then assessed the occurrence of ferroptosis in the fibrotic mouse kidneys. Given that ferroptosis is initiated by the buildup of lipid peroxidation products, and GSH acts as a potent antioxidant by scavenging free radicals and diminishing reactive oxygen species (ROS), while MDA, another lipid peroxidation byproduct, serves as an indicator of lipid peroxidation and ferroptosis, the following strategies were performed. As shown in Figures 3E, F, the fibrotic kidney showed a reduction in GSH levels and an increase in MDA levels, compared with control group. TEM analysis of RTECs revealed mitochondrial atrophy, heightened mitochondrial membrane density, and reduced mitochondrial ridges in the UUO group, indicative of the presence of ferroptosis (Figure 3G).

GPX4, a widely recognized ferroptosis-associated molecule, and HSPA5 were detected in the fibrotic kidneys of mice. IHC assay revealed that GPX4 and HSPA5 were predominantly located in TECs. The GPX4 protein expression showed a significant decrease, whereas HSPA5 levels were elevated in the fibrotic tissues (Figure 3D). Parallelly, the results of Western blotting indicated a similar trend in protein levels of GPX4 and HSPA5 in correlation with IHC following UUO treatment. Moreover, these elevations were time-dependent manner (Figure 3H). Of note, the level of HSPA5 demonstrated a positive correlation with the renal injury score and a negative correlation with GPX4 expression (Figures 3I, J). Overall, these findings strongly indicated that HSPA5 levels and ferroptosis were related to fibrotic kidney, indicating a potential association between HSPA5 and ferroptosis in the formation of kidney fibrosis.

### 3.4 HSPA5 mediated TGF-β1-induced ferroptosis and EMT *in vitro*

**FIGURE 4:**
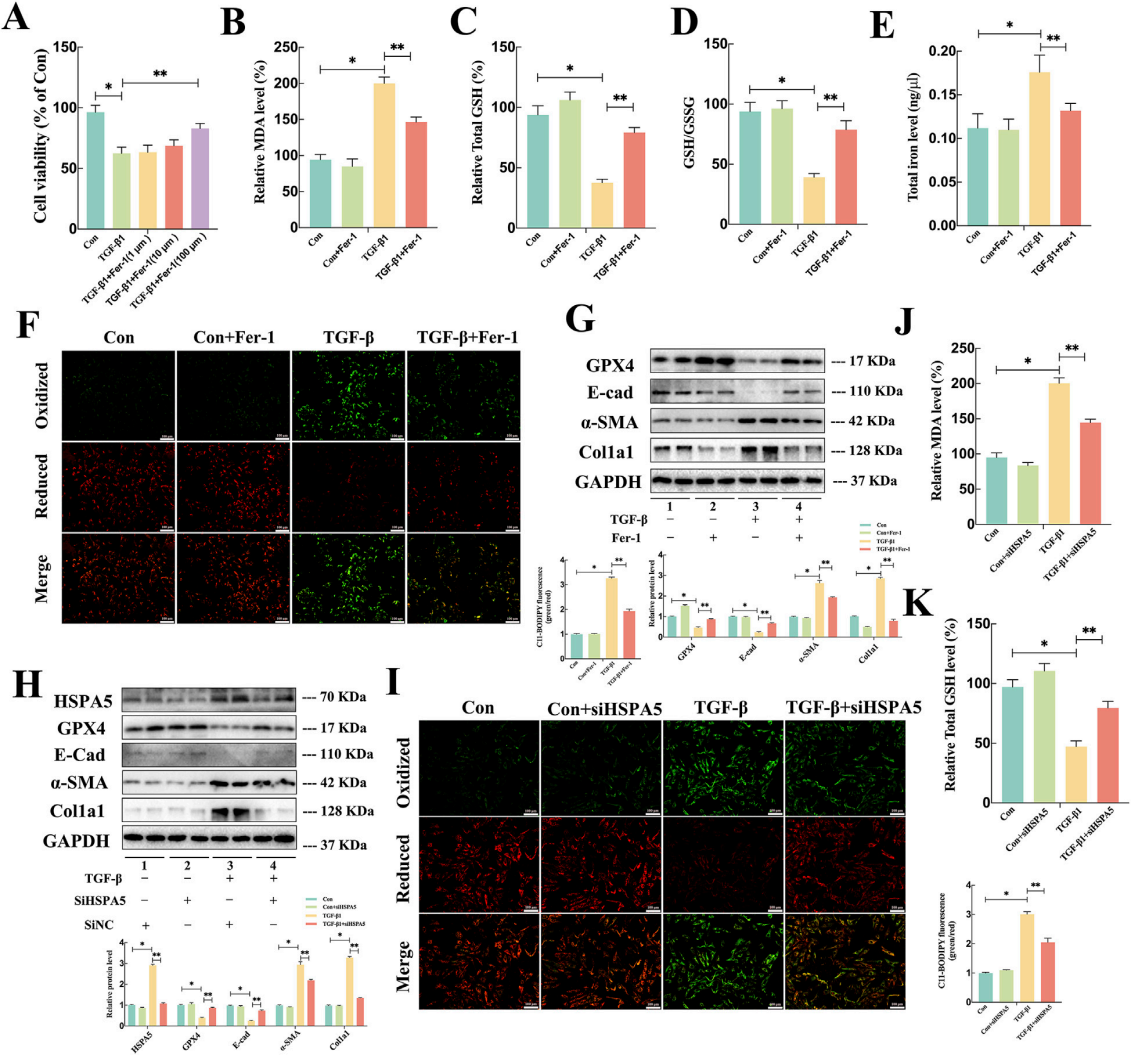
HSPA5 mediated TGF-β1-induced Ferroptosis and EMT in HK-2 cells. **(A)** Cell viability measured via an MTT assay exhibited a noticeable elevation in cell death of HK-2 cells exposed to TGF-β1 (10 ng/mL) in a dose-dependent manner of Fer-1. **(B–E)** The MDA, GSH, GSSG, and iron level were detected in each group (n = 5, *P < 0.05, **P < 0.05) **(F, I)** C11-BODIPY 581/591 was applied to detect lipid peroxidase in HK-2 cells treated with TGF-β1 in combination with Fer-1. **(G–H)** Representative blots of Col1a1, E-cad, α-SMA, GPX4 and HSPA5 expression (n = 5). **(J–K)** The GSH and MDA levels were detected in each group (n = 5, *P < 0.05, **P < 0.05). The results are expressed as the mean ± standard error of the mean (S.E.M.).

To investigate the potential involvement of ferroptosis in TGF-β1-induced EMT of RTECs, Fer-1, a recognized ferroptosis inhibitor, was utilized on HK-2 cells. The administration of Fer-1 significantly improved the reduction in cell viability of TGF-β1-stimulated HK-2 cells in a concentration-dependent fashion (Figure 4A). Since lipid peroxidation and iron accumulation are defining aspects of ferroptosis, we examined lipid metabolism markers including MDA, GSH, GSSG, and iron levels *in vitro*. As illustrated in Figures 4B–E, TGF-β1 induction led to elevated levels of MDA and total iron, accompanied by a reduction in total GSH and the GSH/GSSG ratio in HK-2 cells. It is worth noting that Fer-1 treatment markedly reversed the alterations in these lipid metabolism products caused by TGF-β1. Concurrently, C11-BODIPY 581/591 probe was employed to assess the abundance of lipid peroxides, with fluorescence transitioning from red to green signaling the presence of lipid peroxidation. The results of C11-BODIPY staining analysis indicated that Fer-1 alleviated the elevation of lipid peroxides induced by TGF-β1 (Figure 4F). Moreover, the Western blot analysis results demonstrated that Fer-1 could suppress the production of col1a1 and α-SMA induced by TGF-β1 while also reversing the reduction of GPX4 and E-cad in HK-2 cells (Figure 4G). These results indicated that the ferroptosis contributed to TGF-β1-induced EMT in HK-2 cells.

To further explore critical role of HSPA5-mediated ferroptosis in TGF-β1-induced EMT, HK-2 cells were treated with HSPA5 siRNA. The efficacy of HSPA5 knockdown was confirmed through WB analysis (Figure 4H). As anticipated, TGF-β1 stimulation elevated the levels of HSPA5, triggering ferroptosis and EMT. Conversely, HSPA5 knockdown promoted the expression of E-cad and GPX4, which were associated with ferroptosis, while inhibiting the production of col1a1 and α-SMA in HK-2 cells (Figure 4H). Furthermore, C11-BODIPY staining analysis demonstrated that siHSPA5 mitigated the TGF-β1-induced lipid peroxides increasing (Figure 4I). Moreover, inhibiting HSPA5 reversed the alterations in MDA and total GSH levels induced by TGF-β1 in HK-2 cells (Figures 4J, K). Therefore, these findings indicated that HSPA5 facilitated TGF-β1-stimulated EMT by promoting ferroptosis of RTECs.

### 3.5 ERS inhibition reduced the vulnerability of fibrotic mice to ferroptosis *in vivo*

**FIGURE 5:**
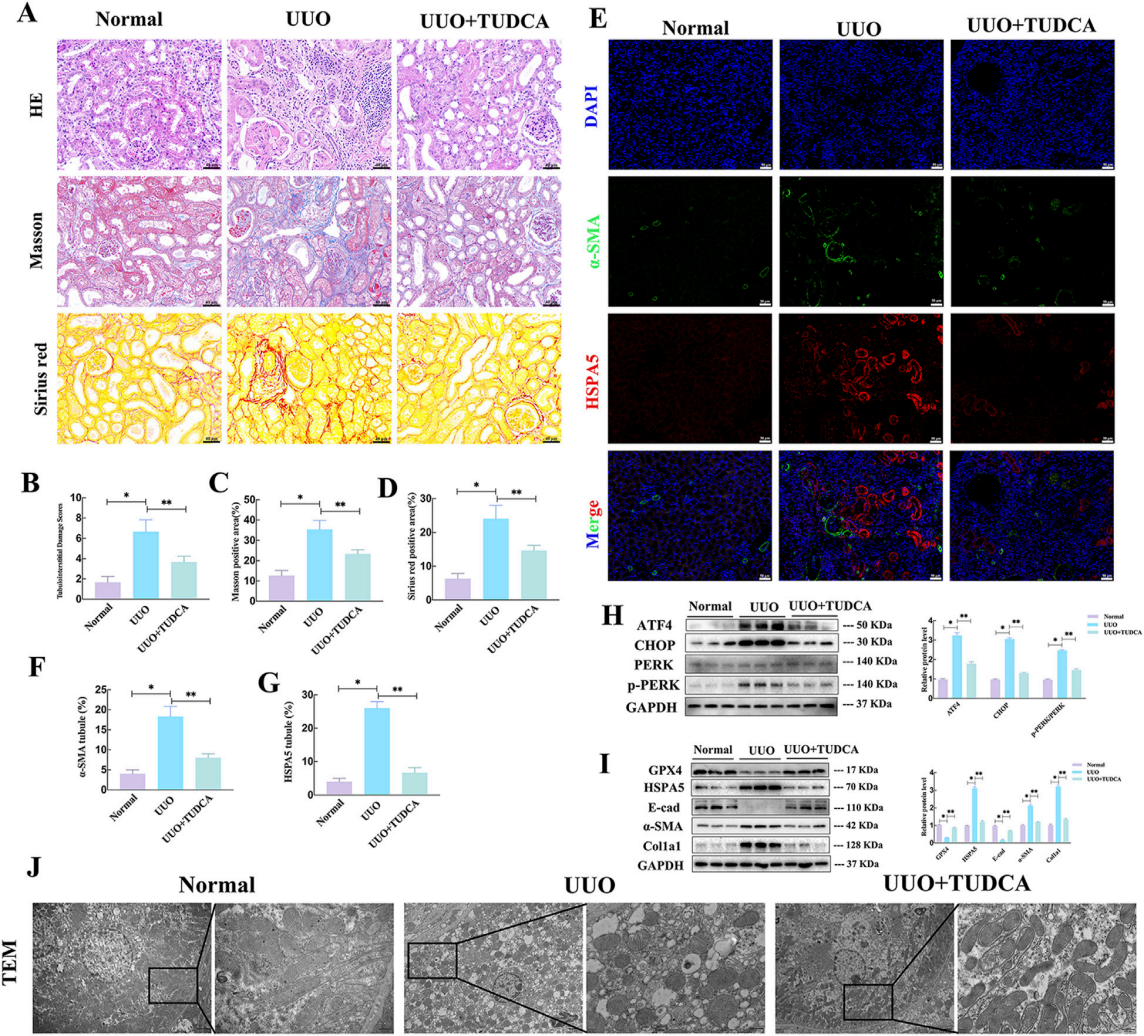
ERS inhibition reduced the vulnerability of fibrotic mice to ferroptosis *in vivo*. **(A)** HE, Masson trichrome and Sirius red staining of kidney interstitium tissue of the 2 groups (Scale bar, 100μm, 20 μm). **(B–D)** Tubulointerstitial damage scores and proportion of positive area were assessed in different groups (n = 6, *P < 0.05, **P < 0.05). **(E–G)** Representative images and statistical analysis of immunofluorescence staining of HSPA5 and α-SMA. **(H–I)** Representative blots of ATF4, CHOP, PERK, p-PERK, Col1a1, E-cad, α-SMA, GPX4 and HSPA5 expression (n = 6). **(J)** Transmission electron microscope images of mitochondria. The results are expressed as the mean ± standard error of the mean (S.E.M.).

Based on prior research, the impact of ERS on ferroptosis varies depending on the context (Xu et al., 2020; Tang et al., 2021). Tauroursodeoxycholate (TUDCA), a traditional inhibitor of endoplasmic reticulum stress (ERS), was used to gain understanding of its involvement in ferroptosis in renal fibrosis. H&E staining revealed higher levels of glomerulosclerosis, tubular injury, and moderate interstitial inflammatory cell infiltration in the UUO group than in the NC group (Figures 5A, B). Masson and Sirius red staining revealed collagen fiber accumulation and increased mesangial matrix in UUO mice (Figures 5A, C, D). Of note, in response to UUO, TUDCA treatment significantly reduced collagen deposition, inhibited tubular necrosis and tubulointerstitial inflammation (Figures 5A–D). Additionally, co-immunofluorescence assays assay manifested that TUDCA treatment reduced the positive staining for HSPA5 in tubular cells and α-SMA in interstitium (Figures 5E–G). Furthermore, the levels of ERS markers and molecules associated with EMT were examined to determine whether exposure to UUO triggered ERS while EMT was advancing. In the UUO group, there was a notable increase in the protein levels of ATF4, p-PERK, and CHOP, suggesting the initiation of ERS. Notably, the changes in EMT-associated molecules caused by UUO, such as the reduction in E-cad levels and the rise in α-SMA and Col1a1 levels, were reversed after TUDCA administration (Figures 5H, I). Interestingly, UUO-stimulated alterations associated with ferroptosis, including GPX4 and HSPA5, were mitigated through TUDCA intervention, indicating a correlation between ERS blockade and ferroptosis (Figures 5H, I). In line with this, TEM studies revealed that the mitochondrial shrinkage, higher mitochondrial membrane concentration, and reduced mitochondrial crests in the kidneys were greatly improved after TUDCA treatment (Figure 5J). Collectively, these findings demonstrated that blocking ERS reduced ferroptosis in UUO mice, accompanied by alleviated EMT progression.

### 3.6 ERS and ferroptosis engaged in crosstalk via ATF4 interacted with HSPA5 interaction *in vitro*

**FIGURE 6:**
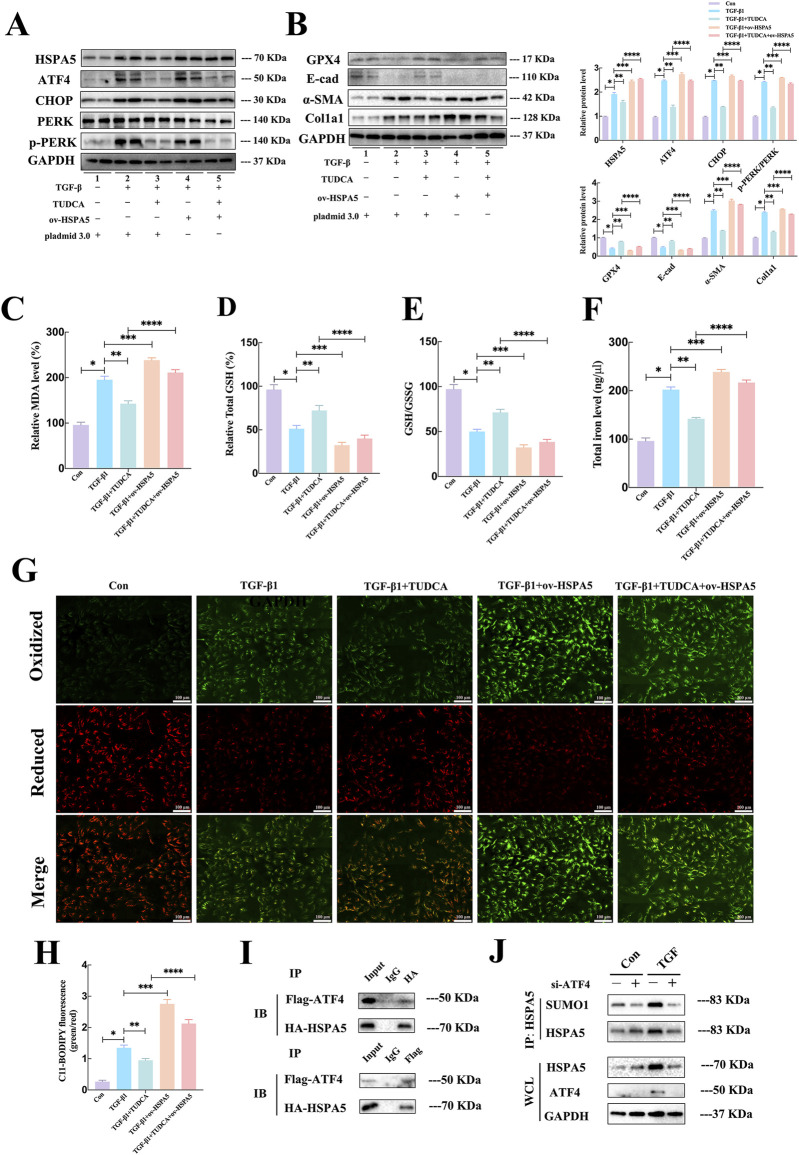
ERS and ferroptosis engaged in crosstalk via ATF4 interacted with HSPA5 interaction *in vitro*. **(A, B)** Representative blots of ATF4, CHOP, PERK, p-PERK, Col1a1, E-cad, α-SMA, GPX4 and HSPA5 expression (n = 6). **(C–F)** The MDA, GSH, GSSG, and iron level were detected in each group (n = 5, *P < 0.05, **P < 0.05). **(G, H)** C11-BODIPY 581/591 was applied to detect lipid peroxidase in HK-2 cells treated with TGF-β1 in each group (n = 5, *P < 0.05, **P < 0.05, ***P < 0.05). **(I)** In HEK293T cells, after co-transfection of Flag-ATF4 and HA-HSPA5, IP assays were performed to enrich Flag-ATF4 and HA-HSPA5. **(J)** IP assays and Western blot to detect the SUMOylation of HSPA5 with ATF4 attenuation.

To delve deeper into understanding the regulatory role and mechanism of ferroptosis following ERS activation, the HK-2 cell line overexpressing HSPA5 was utilized. Consistent with the findings *in vivo*, TUDCA treatment could ameliorate ERS-related proteins expression induced by TGF-β, whereas HSPA5 had minimal impact on endoplasmic reticulum stress (Figure 6A). Additionally, the Western blotting analysis shown that the alterations in EMT-associated molecules caused by TGF-β, including the reduced E-cad expression and elevated α-SMA and Col1a1 expression, were reversed following TUDCA administration. In contrast, HSPA5 overexpression not only exacerbated the progression of TGF-β-stimulated EMT, but partially abolished the protective effects of TUDCA in cell transdifferentiation (Figure 6B). Regarding the ferroptosis, pretreatment of HK-2 cells with TUDCA followed by a 24-hour exposure to TGF-β resulted in the repression of MDA levels (Figure 6C), elevation of GSH levels (Figure 6D), a decrease in GSH/GSSG ratio (Figure 6E), and a reduction in iron levels (Figure 6F). Consistently, the C11-BODIPY staining further confirmed that the TUDCA partially inhibited the lipid peroxidation induced by TGF-β (Figures 6G, H). Predictably, HSPA5 overexpression strikingly attenuated the inhibitory effects of TUDCA on ferroptosis, including GPX4 levels and lipid peroxide buildup (Figures 6B–H), indicating the modulation of ferroptosis by ERS in TGF-β-induced EMT progress is mediated by HSPA5.

Upon encountering ERS, ATF4 undergoes selective translation to increase the production of substances that promote the survival of cancer cells under stressful conditions (Zheng et al., 2023). Given that HSPA5 served as a key chaperone that could be increased by ATF4 under ERS (Luo et al., 2003), we investigated the influence of ATF4-HSPA5 interaction on HSPA5. Firstly, we generated the overexpressing plasmids HA-HSPA5 and Flag-ATF4, followed by their co-transfection into the HEK293T cell line. The exogenous interaction was confirmed through an immunoprecipitation (IP) assay (Figure 6I). The regulation of protein function within cells is heavily influenced by post-translational modifications (PTMs), hence we put forward our hypothesis whether ATF4 could mediated the PTMs of HSPA5. To validate the hypothesis, the quantified immunoprecipitation (qIP) assay demonstrated that ATF4 silence markedly suppressed TGFβ-induced SUMOylation of HSPA5 (Figure 6J). These finds indicated that ATF4 interacted with and SUMOylated HSPA5 to activate HSPA5 signaling pathway, resulted in the activation of ferroptosis.

## 4 Discussion

This study elucidated a critical role of ERS-regulated HSPA5 in renal fibrosis through a comprehensive analysis of genetic and pharmacological experiments. The primary discoveries are as follows: (i) HSPA5 expression was markedly increased in the TECs of fibrotic kidneys, concurrent with ERS and ferroptosis in UUO-mice; (ii) HSPA5 served as a crucial intermediary in regulating ferroptosis under ERS in renal fibrosis through the utilization of ERS specific inhibitor and ATF4/HSPA5-knockin/-knockdown cellular model. (iii) Mechanistically, ATF4 interacted with HSPA5, leading to increased SUMOylation of HSPA5 and exacerbated HSPA5-mediated ferroptosis in TGF-β-induced EMT. The findings clarify the processes at play and suggest that focusing on HSPA5 may be a viable treatment approach to improve the longevity of tubular epithelial cells and reduce renal fibrosis in individuals with CKD.

TEC, a major source of myofibroblasts, was found to be essential in driving the advancement of renal fibrosis via EMT process (Hong et al., 2019). Overactivation of oxidative stress reactions contributing to the advancement of EMT in renal TECs of CKD patients could lead to various forms of cell demise, such as necrosis, apoptosis, and ferroptosis (Shen et al., 2022). Ferroptosis is a form of programmed cell death that relies on iron, often characterized by iron buildup and lipid peroxidation. This process is complex and characterized by reduced ability to fight off antioxidants and buildup of lipid ROS (Jiang et al., 2021). In CKD, dysfunction of iron regulatory proteins results in iron accumulation and oxidative damage. The deposition of iron is influenced by both external iron supplementation and internal iron-driven reactions triggered by ischemia and hypoxia (Staniek and Wojciak, 2018). In a mouse CKD model induced by UUO, HO-1 deletion upregulated TGF-β1 expression, exacerbating renal fibrosis, with ferroptosis inhibitor, preventing renal tubulointerstitial fibrosis by regulating TGF-β1-Smad signaling, oxidative stress, and the inflammatory response (Han et al., 2024). Zhang et al. also showed that blockage of the ureter caused ferroptosis in TECs in a living organism, leading to the release of various fibrosis-promoting substances that then affected nearby interstitial fibroblasts, boosting the growth and specialization of these cells. (Zhang et al., 2021). In line with the previous studies, proteins associated with ferroptosis, such as GPX4, was downregulated, resulting in the vanishing of mitochondrial cristae and breakage of the outer mitochondrial membrane in TECs. Nevertheless, the influence of ferroptosis on the progression of renal tubular EMT in CKD is still unclear. This study revealed that treating with Fer-1 reversed the increase in α-SMA and Col1a1 levels, decreased E-cadherin levels, and reduced mesangial matrix expansion and collagen fiber deposition caused by TGF-β *in vitro*, suggesting that ferroptosis contributed to the EMT process and worsened renal fibrosis in CKD.

Our bioinformatics analysis further revealed diverse expression profiles of ferroptosis regulators in CKD. Specifically, HSPA5 emerged as a pivotal regulator of ferroptosis, exhibiting associations with the overall prognosis of CKD. HSPA5 was known to participate in numerous cancers; however, the diverse levels of its expression exhibit substantial variations in the prognosis and advancement of distinct cancer types. Wang et al. reported that HSPA5 inhibition heightened the sensitivity of breast cancer cells to ferroptosis through the P53/SLC7A11/GPX4 pathway (Wang Q. et al., 2023). A group led by Yuan Wang demonstrated that the acetylation of HSPA5 by EP300 amplified the HSPA5-induced suppression of GPX4, leading to heightened lipid peroxidation and ferroptosis in pancreatic cancer (Wang Y. et al., 2023). Shu et al. demonstrated the persistent ER stress, mediated by HSPA5, in post-AKI kidneys, which was involved in the development of chronic renal pathologies and CKD (Shu et al., 2018). Whereas the research on the role of HSPA5 in non-neoplastic diseases was relatively scarce. In our research, although the HSPA5 expression was markedly elevated in renal fibrosis, resembling that in cancer diseases, its function was entirely distinct. Herein, we showed that inhibiting HSPA5 reversed the alterations in MDA, total GSH levels and EMT-related proteins expression treated with TGF-β1 *in vitro*. These results demonstrated that HSPA5 was a potential therapeutic target for renal fibrosis, different from the multiple cancers, and served as an effective predictive biomarker. Overall, HSPA5 suppresses ferroptosis in cancer cells while facilitating tumor cell migration and metastasis. In the context of CKD, HSPA5 contributes to the renal tubular EMT by inducing ferroptosis in TECs, leading to an exacerbation of fibrosis. Further investigation is necessary to delve into the fundamental mechanisms behind these differences.

Accumulation of lipids inside cells and the presence of oxidative stress can disturb the balance of the endoplasmic reticulum, leading to ERS, ultimately triggering ferroptosis (Xu et al., 2020; Lebeaupin et al., 2018). A recent report by Zijun Liu (Liu et al., 2023) showed that ERS initiated the progression of ferroptosis-linked EMT via the XBP1-Hrd1-Nrf2 pathway in diabetic kidney disease. Herein, TUDCA therapy reduced the accumulation of collagen fibers, which were key indicators of EMT in UUO mice. Significantly, the pharmacological blockade of ERS effectively reversed the alterations associated with ferroptosis, including iron accumulation, buildup of lipid peroxidation products and the reduction of GPX4 expression in the renal tissues of UUO mice and TGF-β-exposed HK-2 cells. In addition to its significant linkage with ferroptosis, HSPA5 acted as a molecular chaperone of HSP70 within the ER, playing a role in protein folding and assembly and governing ER homeostasis (Ibrahim et al., 2019). Jin R. and colleagues reported that HSPA5 participated in ERS by binding with S100 calcium-binding protein 16 and stimulating IRE1α (Jin et al., 2021). Nevertheless, its role and mechanism in renal fibrosis have yet to be elucidated. In this study, the overexpression of HSPA5 strikingly attenuated the inhibitory effects of TUDCA on ferroptosis and TGF-β-induced EMT *in vitro*, indicating the modulation of ferroptosis by ERS in TGF-β-induced EMT progress is mediated by HSPA5. Being a crucial transcription factor in ERS precess, the expression of ATF4 is specifically enhanced by phosphorylated through the phosphorylation of eIF2α triggered by PERK activation (Wortel et al., 2017). When exposed to ERS, ATF4 selectively undergoes translation to boost the expression of molecules that support the adaptive survival of cancer cells, notably SLC7A11/xCT3 (Zheng et al., 2023). Furthermore, the PERK-mediated activation of ATF4 has demonstrated protective functions to inhibit ferroptosis of cancer cells (Dixon et al., 2014). Given that HSPA5 served as a primary chaperone that could be upregulated by ATF4 under ERS, we examined the influence of ATF4-HSPA5 interaction on HSPA5. Our study revealed that the ATF4-mediated SUMOylation of HSPA5 enhanced HSPA5’s inhibition of GPX4, leading to elevated lipid peroxidation, facilitating ferroptosis, and contributing to EMT. These finds indicated that ATF4 interacted with and SUMOylated HSPA5 to activate HSPA5 signaling pathway, resulted in the activation of ferroptosis.

## 5 Conclusion

In conclusion, this research highlights the substantial involvement of HSPA5 in mediating ferroptosis, its role in the progression of EMT in renal fibrosis, and the interconnected relationship between ERS, a catalyst for ferroptosis, in the development of renal interstitial fibrosis. Importantly, ERS has the potential to induce ferroptosis-associated EMT through the SUMOylation of HSPA5 mediated by ATF4. The results shed light on the processes and suggest that focusing on HSPA5 could be a beneficial treatment strategy to improve the survival of tubular epithelial cells and reduce the advancement of CKD.

## Data Availability

The raw data supporting the conclusion of this article will be made available by the authors, without undue reservation.
